# Dissociable effects of cocaine and yohimbine on impulsive action and relapse to cocaine seeking

**DOI:** 10.1007/s00213-017-4711-9

**Published:** 2017-08-30

**Authors:** Nienke Broos, Yvar van Mourik, Dustin Schetters, Taco J. De Vries, Tommy Pattij

**Affiliations:** Department of Anatomy and Neurosciences, Amsterdam Neuroscience, VU University Medical Center, De Boelelaan 1108, 1081 HZ Amsterdam, The Netherlands

**Keywords:** Impulsivity, Cocaine self-administration, Relapse, Yohimbine

## Abstract

**Rationale:**

A strong association has been demonstrated between various forms of impulsivity and addiction-like behavior in both humans and rats.

**Objectives:**

In this study, we investigated how impulsive action, as measured in the 5-choice serial reaction time task (5-CSRTT), is affected during various stages of cocaine taking and seeking and by relapse-provoking stimuli in animals that were trained both in an intravenous cocaine self-administration paradigm and in the 5-CSRTT.

**Methods:**

Rats were concurrently trained in the 5-CSRTT and cocaine self-administration protocol, and subsequently, the effects of cocaine (7.5 mg/kg) and the pharmacological stressor yohimbine (1.25 mg/kg) were tested in both paradigms.

**Results:**

Cocaine self-administration (5 h/day) transiently altered impulsive action and increased errors of omission in the 5-CSRTT. Pharmacological challenges with cocaine and yohimbine induced increments in impulsive action and reinstated cocaine-seeking responses within the same animals. Further analyses revealed that the effects of cocaine and yohimbine on impulsive action did not correlate with their effects on reinstatement of cocaine seeking.

**Conclusions:**

These data suggest that although impulsive action and relapse can be pharmacologically modulated in the same direction within individuals, these effects appear not to be directly coupled.

## Introduction

There is broad consensus that drug dependence and impulsivity are closely related. For instance, clinically there is high comorbidity between attention-deficit/hyperactivity disorder (ADHD) and substance-use disorders (Van Emmerik-van Oortmerssen et al. 2012). Maladaptive impulsivity is a key symptom in ADHD (Moeller et al. 2001) and, moreover, elevated impulsivity is also frequently observed in substance dependence, including cocaine dependence (for reviews, see De Wit 2009; Verdejo-Garcia et al. 2008; Pattij and De Vries 2013). Additionally, preclinical animal studies have unequivocally demonstrated that impulsive rats are more vulnerable to several measures of addiction-like behavior (Belin et al. 2008; Diergaarde et al. 2008; Dalley et al. 2007; Broos et al. 2015; Jupp et al. 2013; Perry and Carroll 2008; Winstanley et al. 2010).

Impulsivity is widely viewed as action without adequate forethought and consists of dissociable behavioral modalities (Broos et al. 2012b; Evenden 1999; Winstanley et al. 2004). These dissociable modalities of impulsivity show distinct relationships with various phases of drug addiction (for reviews, see Winstanley et al. 2010, Pattij and De Vries 2013). One of these modalities, impulsive action, arises from deficient inhibitory-response control and relates to an elevated risk to the escalation of compulsive cocaine intake (Belin et al. 2008; Dalley et al. 2007). In addition, high levels of impulsive action are associated with a stronger cue-induced reinstatement response after punishment-induced abstinence (Economidou et al. 2009). Together these studies highlight that impulsivity co-exists with high risk to addictive-like behavior, yet do not necessarily imply that impulsivity mediates this risk.

One important mediator of relapse in drug dependence is stress, and over the last two decades, translational models have tremendously contributed to our understanding of the neural correlates of stress-induced relapse (for a recent review, see Mantsch et al. 2016). A variety of stressors are able to reinstate drug seeking, among which is the pharmacological stressor yohimbine (Charney et al. 1984; Shepard et al. 2004). Besides cues and stress, relapse to cocaine seeking can be reliably provoked by priming injections of cocaine (for a review, see Bossert et al. 2005). Interestingly, cocaine and yohimbine have also been demonstrated to induce an increase in impulsive action (Schippers et al. 2016; Sun et al. 2010; Torregrossa et al. 2012; van Gaalen et al. 2006; Winstanley et al. 2007). Despite the fact that yohimbine and cocaine have similar effects on relapse propensity and impulsive action, it remains to be studied whether overlapping mechanisms are responsible for this, or whether independent mechanisms underlie these effects.

The present study aims to explore the interrelationship between impulsive action and cocaine relapse by continuous monitoring of impulsivity levels during various stages of long-access cocaine taking and seeking. Additionally, the acute effects of cocaine and yohimbine on impulsive action and reinstatement of cocaine seeking were directly compared using a within-subject design.

## Materials and methods

### Animals

Male Wistar rats (Harlan, Horst, The Netherlands), initially weighing 250–270 g, were housed in standard Macrolon cages on a reversed 12-h day/night cycle (lights on, 7 p.m.) in a temperature (21 ± 2 °C) and humidity (50 ± 10%) controlled room. Rats were housed in pairs until surgery, and individually afterwards. Behavioral testing was conducted during the dark phase of the day/night cycle. During the entire experiment, rats were food restricted and maintained at about 85–90% of their free-feeding weight by providing them with 14–18 g of chow at the end of each day. Water was available ad libitum. Experiments were approved by the Animal Care committee of the VU University and VU University medical center, Amsterdam, The Netherlands.

### Five-choice serial reaction time task (5-CSRTT)

Detailed descriptions of apparatus and training procedures have been provided previously (Van Gaalen et al. 2006). In short, the final procedure of the 5-CSRTT was as follows: rats were placed in an operant chamber containing a food receptacle and an array of five rectangular apertures in the opposing wall. After starting the trial by a nose poke in the receptacle, they were required to wait for 5 s (inter-trial interval, ITI) before one of the stimulus lights within the apertures was illuminated for 1 s. A nose-poke response into this illuminated hole was rewarded with one food pellet. Every session consisted of 100 trials or lasted 30 min, whichever occurred first. The following behavioral measures were recorded to assess task performance: (1) accurate choice, i.e., percentage correct responses calculated as [number correct trials/(correct + incorrect trials)] × 100 as a measure of visuospatial attention; (2) premature responses as a measure of impulsive action, i.e., number of responses into any of the holes during the ITI period and before stimulus onset; (3) number of omissions, i.e., number of omitted trials during a session as a measure of motivation and/or motor performance; (4) perseverative responses after correct choice, as a measure of inhibition related to compulsive-like response patterns; (5) correct response latency, i.e., the mean time between stimulus onset and a correct response as a measure of speed of responding and/or motor performance; and (6) feeder latency, i.e., the latency to collect a pellet following a correct choice as a measure of motivation.

### Cocaine self-administration

Detailed description of apparatus and training procedures has been provided previously (Broos et al. 2012a). Briefly, operant two-lever chambers were equipped with a red house light, a white noise generator, and a liquid swivel connecting rats to an infusion pump (total volume of 42.52 μl delivered over 2 s). Rats were randomly assigned to one of two different contexts. These contexts differed in (1) white noise (70 dB), either continuous or interval; (2) odor, either lemon-scented or almond-scented; and (3) the chamber floor, a flat PVC surface with either holes or straight grooves. The employed cocaine-taking and cocaine-seeking paradigm started with acquisition of cocaine (cocaine-HCl, OPG, Utrecht, The Netherlands) self-administration under a fixed-ratio 1 (FR1) schedule of reinforcement and occurred in daily 5-h sessions in the designated context. To reduce the anxiogenic effects of cocaine, rats received 250 μg/kg/infusion of cocaine during the first five sessions, followed by 500 μg/kg/infusion for the remaining sessions. To prevent overdosing in inexperienced rats, the maximum number of rewards was set to 40 rewards for the first three sessions and to 100 rewards for the next two sessions. After this, animals could earn an unlimited number of infusions. There was a non-signaled time-out of 15 s after every infusion. Inactive lever presses were registered, but without consequences. Subsequently, following acquisition, responding under a progressive ratio schedule of reinforcement was assessed in four subsequent identical 4-h sessions to assess the motivation to self-administer cocaine. During progressive ratio responding for cocaine, the response ratio requirement and number of active responses resulting in a cocaine infusion was progressively increased between infusions following the equation response ratio = 5 × *e* ^(0.2 × infusion number)^ − 5, rounded to the nearest integer.

Extinction of cocaine self-administration was measured in two different types of extinction procedures. First, to test the effects of exposure to the drug-associated context on reinstatement of cocaine seeking, operant extinction training took place to extinguish responses on the active lever. This extinction procedure consisted of daily 1-h sessions in a context different from the cocaine SA context, in which no odor was introduced, white noise was absent, and there was a grid floor. During operant extinction training, all responses were registered, but without programmed consequences. Following operant extinction, a context-induced reinstatement test was conducted by exposing animals to the cocaine-associated context to determine whether this would elicit drug-seeking responses. Subsequently, a second extinction procedure was followed by subjecting the animals to context extinction to allow assessment of the effects of cocaine and yohimbine on reinstatement of drug-seeking responses. During context-extinction sessions which lasted 1 h, the cocaine-associated context was reintroduced into the operant chamber (white noise, odor, and PVC floor). All responses were registered, but without consequences. Following context extinction, a cocaine-induced reinstatement test was performed under context-extinction conditions that lasted 30 min. During this reinstatement test, 20 min prior to testing, all rats received intraperitoneal injections of saline (1 ml/kg bodyweight) on the first test day and cocaine (7.5 mg/kg) on the following test day. Subsequently, five additional context-extinction sessions were given followed by a stress-induced reinstatement test using yohimbine as a pharmacological stressor. For this, rats received an acute challenge with the α2-adrenoceptor antagonist yohimbine (1.25 mg/kg, intraperitoneal, 1 ml/kg bodyweight; Sigma, St. Louis, MO, USA) 45 min prior to the reinstatement test. Rats received sterile water (1 ml/kg bodyweight) on the first test day and yohimbine on the following test day.

### Experimental design

**Fig. 1:**
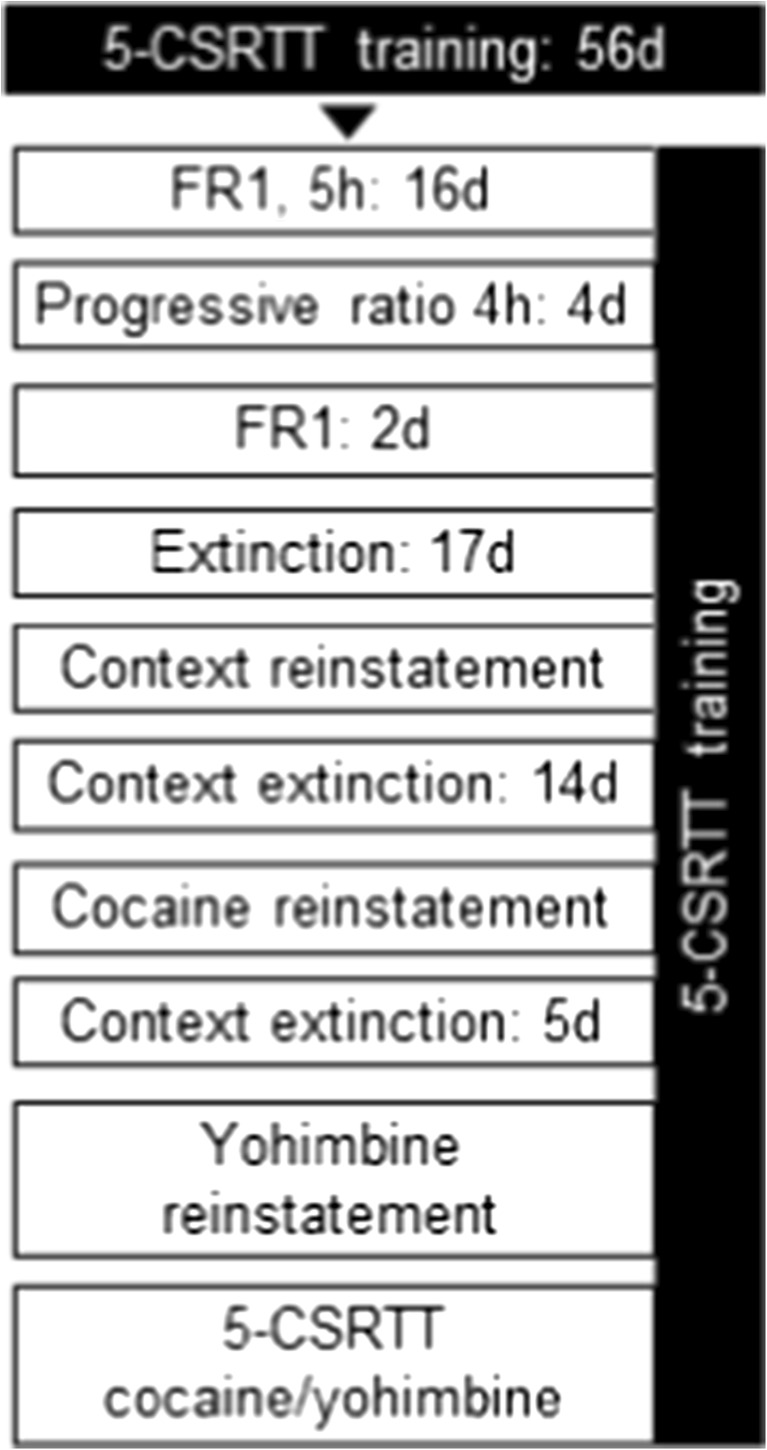
Schematic diagram depicting the design and order of the experiments

The current experiment was designed to explore whether the co-occurrence between impulsive action and relapse to cocaine seeking is due to interrelated phenomena. To that end, we compared direct manipulations of impulsive action with direct manipulations of reinstatement to drug seeking in the same animals using a within-subject design (Fig. 1). A cohort of 32 rats was trained in the 5-CSRTT and upon stable baseline performance equipped with jugular catheters. Upon reestablishment of stable 5-CSRTT performance, all rats were trained in the afternoon to self-administer cocaine under long-access (5 h) conditions. The following behavioral stages of cocaine taking and cocaine seeking were studied: (1) cocaine self-administration on FR1 schedule (16 sessions), (2) progressive ratio responding (four sessions), (3) operant extinction (17 sessions), (4) context-induced reinstatement, (5) context extinction (14 sessions), and (6) cocaine- and yohimbine-induced reinstatement. Subsequently, 1 week following these tests, the effects of acute cocaine and yohimbine challenges on 5-CSRTT performance (including impulsive action) were tested. First, acute effects of cocaine/saline were tested, and a week later, acute effects of yohimbine/water were tested. In the 5-CSRTT, the drugs and their respective vehicles were tested in counterbalanced fashion within individuals across both testing days which were Tuesdays and Thursdays with baseline training sessions on other weekdays. Dosing and timing of injections were identical to the reinstatement tests. During the entire cocaine SA paradigm, rats were trained in the 5-CSRTT every morning on weekdays, and the presented data of concurrent 5-CSRTT sessions show the performance averaged per week similar to previous work (Winstanley et al. 2009). Due to the loss of catheter patency in the course of the experiments, *n* = 9 animals were excluded, leaving *n* = 23 animals in all analyses unless stated otherwise.

## Statistical analyses

Data were analyzed using repeated measures analysis of variance (ANOVA) and, in the case of the pharmacological challenges, a two-way repeated measures ANOVA with dose (vehicle or dose) and drug (cocaine or yohimbine) as within-subjects variable. For the ANOVAs, the homogeneity of variance was determined using Mauchly’s tests for equal variances, and in the case of violation of homogeneity, Huynh-Feldt epsilon (*ε*) adjusted the degrees of freedom; the resulting more conservative probability values were depicted and used for subsequent analyses. For SA data, sessions and lever (active vs inactive) served as within-subjects variables and the number of active and inactive responses were dependent variables. For the 5-CSRTT data, the dependent variables were the number of premature responses, accuracy, number of omissions, perseverative responses, and correct response and feeder latencies. Pearson’s correlation analyses were used to test whether reactivity to cocaine and yohimbine compared to their respective vehicle in the 5-CSRTT and reinstatement paradigms was related. Data were analyzed using the Statistical Package for the Social Sciences version 20.0 (SPSS, Chicago, IL, USA), and the significance level was set at *p* < 0.05.

## Results

### Cocaine self-administration paradigm

**Fig. 2:**
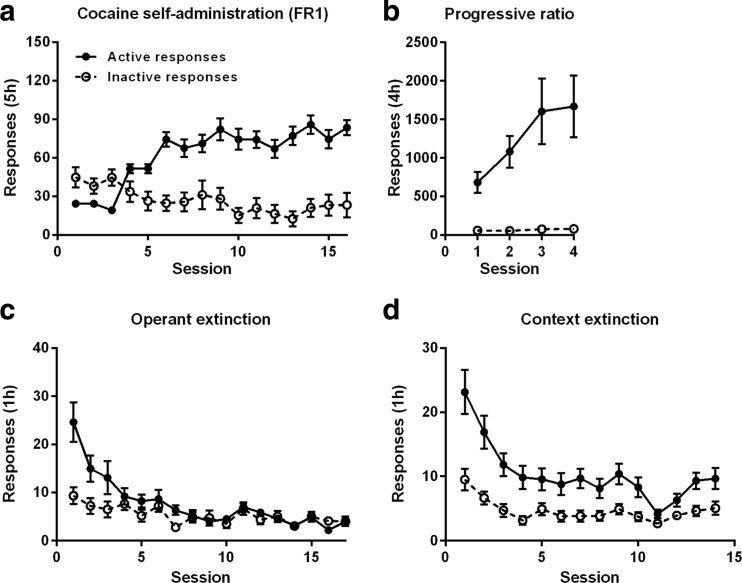
Measures of cocaine taking and seeking behavior in all included *n* = 23 animals. **a** Number of cocaine infusions/responses during cocaine self-administration under an FR1 schedule of reinforcement. **b** Number of responses under progressive ratio responding for cocaine. **c** Number of responses during operant extinction of cocaine seeking. **d** Number of responses during context extinction of cocaine seeking in the cocaine-associated context

All rats readily acquired cocaine self-administration over the course of 16 sessions by increments in active lever responding to the level of approximately 80 cocaine infusions per session, whereas responding on the inactive lever did not significantly change [Fig. 2a; active responses: *F*(15,330) = 29.95, *p* < 0.001, *ε* = 0.36; inactive responses: *F*(15,330) = 1.96, *ε* = 0.50, NS]. Following this, responding for cocaine under a progressive ratio schedule of reinforcement increased the levels of active responding but not the inactive responding over the different ratios during four subsequent days [Fig. 2b; active responses: *F*(3,66) = 3.92, *ε* = 0.54, *p* = 0.037; inactive responses: *F*(3,66) = 1.17, *ε* = 0.68, NS]. After progressive ratio responding, animals were subjected to two additional FR1 sessions (data not shown), and thereafter during 17 subsequent sessions, operant responding for cocaine was extinguished in a neutral context and all rats rapidly decreased responding on both the active lever and inactive lever [Fig. 2c; active responses: *F*(16,352) = 13.90, *ε* = 0.20, *p* < 0.001; inactive responses: *F*(16,352) = 4.03, *ε* = 0.50, *p* < 0.001] in the absence of cocaine. Exposure of the rats to the cocaine-associated context reinstated responding on the active lever that subsequently decreased over 14 sessions of context extinction training to the cocaine-associated context [Fig. 2d; active responses: *F*(13,286) = 11.49, *ε* = 0.42, *p* < 0.001; inactive responses: *F*(16,352) = 4.03, *ε* = 0.50, *p* < 0.001].

### 5-CSRTT performance during the cocaine self-administration paradigm

**Fig. 3:**
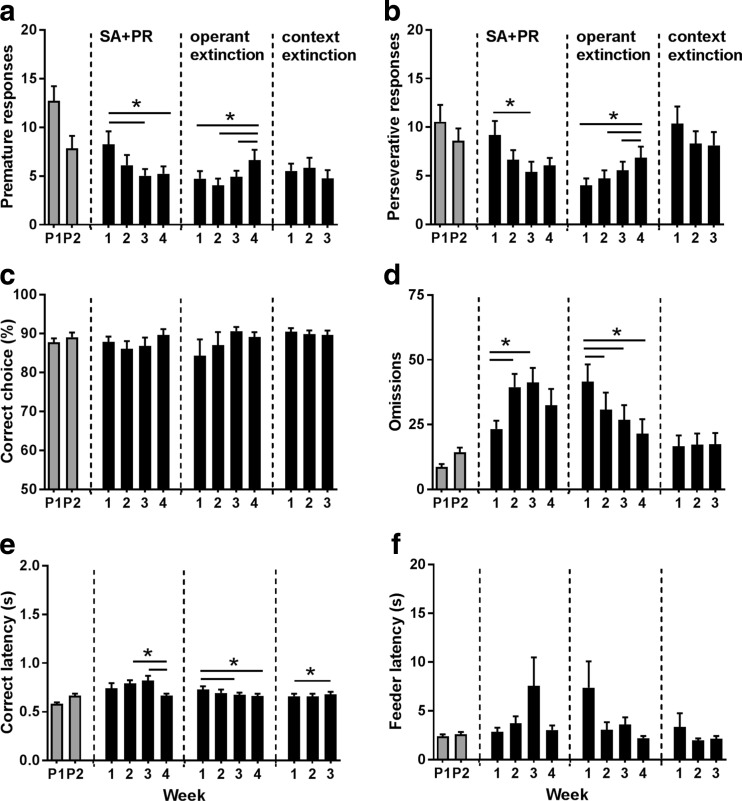
Concurrent training in the 5-CSRTT during cocaine taking and seeking behavior. Impulsive action (**a**) and the number of perseverative responses (**b**) decreased during cocaine self-administration and increased during operant extinction training. Visuospatial attention is not affected during cocaine self-administration and extinction learning (**c**), whereas both omissions (**d**) and response latencies (**e**) increase during cocaine self-administration and decrease during operant extinction training. Feeder latencies (**f**) are not affected during cocaine self-administration and extinction training. P1 and P2 indicate baseline performance levels prior to intravenous catheter surgery (P1) and before cocaine self-administration (P2), respectively. SA+PR indicate self-administration (SA, during all sessions in weeks 1–3) and progressive ratio responding (PR, based on four PR sessions during week 4). *p < 0.05

Concurrent training in the 5-CSRTT during cocaine SA revealed that during acquisition and progressive ratio responding for cocaine, rats became less impulsive in the last two weeks of self-administration compared to the first week [Fig. 3a; *F*(3,66) = 3.53, *ε* = 0.80, *p* = 0.029]. In contrast, over the course of operant extinction learning, premature responses significantly increased during week 4 compared to the other three weeks of the operant extinction procedure [*F*(3,66) = 3.89, *ε* = 0.72, *p* = 0.025], whereas during context extinction, premature responding did not change [*F*(2,44) = 1.70, NS]. Likewise, perseverative responding after correct choice also significantly decreased during week 3 of cocaine SA compared to the first week [Fig. 3b; *F*(3,66) = 3.1, *ε* = 0.73, *p* = 0.048] and increased during weeks 3 and 4 of operant extinction learning compared to week 1 [*F*(3,66) = 5.84, *ε* = 0.76, *p* = 0.004] yet did not change over the course of context extinction [*F*(2,44) = 3.27, *ε* = 0.77, NS]. Levels of accurate choice were neither affected during acquisition and PR responding for cocaine SA [Fig. 3c; [*F*(3,66) = 1.53, *ε* = 0.59, NS] nor during operant extinction learning [*F*(3,60) = 0.94, *ε* = 0.42, NS] and context extinction learning [*F*(2,42) = 1.10, NS]. Importantly, *n* = 2 animals consistently omitted all trials within a 5-CSRTT session over the entire operant extinction learning period. Of these *n* = 2 animals, one animal maintained omitting all trials over the context-extinction period. The number of omitted trials, significantly increased during weeks 2 and 3 compared to week 1 of cocaine SA and progressive ratio responding [Fig. 3d; *F*(3,66) = 5.08, *ε* = 0.82, *p* = 0.006], whereas during operant extinction learning, the number of omissions significantly decreased over weeks [*F*(3,66) = 12.62, *ε* = 0.46, *p* < 0.001], which remained stable over context extinction learning [*F*(2,44) = 0.93, NS]. Response latencies were shortened in week 4 compared to weeks 2 and 3 during cocaine SA and progressive ratio responding [Fig. 3e; *F*(3,66) = 4.92, *ε* = 0.80, *p* = 0.008], and also, during weeks 3 and 4 of operant extinction learning, response latencies were shortened compared to the first week of extinction [*F*(3,60) = 3.18, *ε* = 0.71, *p* = 0.049] and were lengthened during the final week of context extinction compared to the first week [*F*(2,42) = 3.78, *p* = 0.031]. Finally, feeder latencies were altered neither during cocaine SA and progressive ratio responding [Fig. 3f; *F*(3,60) = 2.33, *ε* = 0.38, NS] nor during operant extinction learning [*F*(3,60) = 2.96, *ε* = 0.37, NS] and context extinction learning [*F*(2,44) = 0.37, *ε* = 0.69, NS].

### Cocaine and yohimbine effects on reinstatement to cocaine seeking and 5-CSRTT performance

**Fig. 4:**
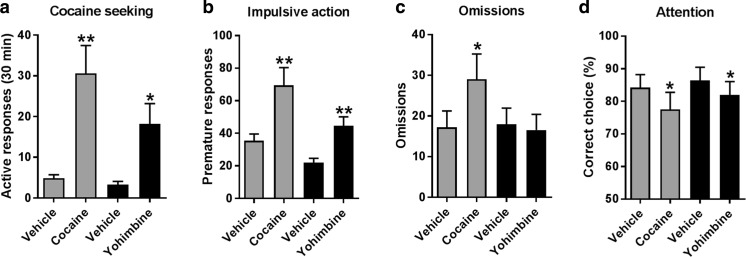
Acute effects of cocaine and yohimbine on reinstatement to cocaine seeking and on 5-CSRTT performance. Both cocaine (7.5 mg/kg) and yohimbine (1.25 mg/kg) induced reinstatement of cocaine seeking (**a**) and increased impulsive action in the 5-CSRTT (**b**). In contrast, only cocaine increased the number of omissions in the 5-CSRTT (**c**), whereas only yohimbine reduced visuospatial attention in the 5-CSRTT (**d**). *p < 0.05 and **p < 0.005 compared to respective vehicle

Following the context-induced reinstatement test and extinction of the cocaine-associated context, acute effects of 7.5 mg/kg cocaine and 1.25 mg/kg yohimbine were tested on reinstatement of cocaine seeking. During these tests, both cocaine and yohimbine strongly increased the number of active lever responses [Fig. 4a; dose: *F*(1,22) = 20.85, *p* < 0.001; drug: *F*(1,22) = 3.84, NS; drug × dose: *F*(1,22) = 2.37, NS]. In addition, the number of inactive responses during reinstatement were also increased by cocaine from 1.9 (± 0.5) to 4.3 (± 1.6) responses and by yohimbine from 2.1 (± 0.7) to 8.0 (± 2.5) responses [data not shown; dose: *F*(1,22) = 12.21, *p* = 0.002; drug: *F*(1,22) = 1.23, NS; drug × dose: *F*(1,22) = 1.42, NS].

Following reinstatement tests, in the 5-CSRTT acute challenges with 7.5 mg/kg cocaine and 1.25 mg/kg yohimbine, both strongly increased the number of premature responses compared to their respective vehicles with higher response rates in the cocaine experiments [Fig. 4b; dose: *F*(1,22) = 28.43, *p* < 0.001; drug: *F*(1,22) = 9.41, *p* = 0.006; drug × dose: *F*(1,22) = 1.42, NS]. Whereas cocaine increased the number of omissions, this measure was not affected by yohimbine [Fig. 4c; dose: *F*(1,22) = 3.71, NS; drug: *F*(1,22) = 4.08, NS; drug × dose: *F*(1,22) = 6.14, *p* = 0.021]. The levels of accurate choice were reduced by both cocaine and yohimbine [Fig. 4d; dose: *F*(1,22) = 6.93, *p* = 0.015; drug: *F*(1,22) = 2.92, NS; drug × dose: *F*(1,22) = 0.29, NS]. Lastly, although the effect size was small, response latencies were reduced from 0.61 (± 0.03) to 0.56 (± 0.04) s by cocaine and from 0.60 (± 0.03) to 0.59 (± 0.03) s by yohimbine [data not shown; dose: *F*(1,22) = 4.49, *p* = 0.046; drug: *F*(1,22) = 0.31, NS; drug × dose: *F*(1,22) = 1.98, NS]. Other measures reflecting task performance in the 5-CSRTT, namely perseverative responses and feeder latencies, were not altered by cocaine or yohimbine [data not shown; all *F* < 1.13, NS].

### Correlation analyses of the reactivity to cocaine and yohimbine in the 5-CSRTT and cocaine reinstatement

**Fig. 5:**
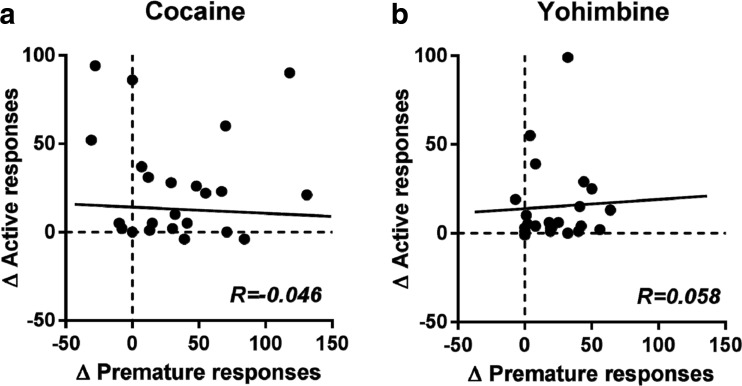
Correlation between reactivity to cocaine and yohimbine compared to their respective vehicle on impulsive action in the 5-CSRTT and reinstatement to cocaine seeking. There was no correlation between the effects of both cocaine (**a**) and yohimbine (**b**) on the number of premature responses in the 5-CSRTT and the number of active cocaine-seeking responses

All correlations between the effects of cocaine and yohimbine on the behavioral measures in the 5-CSRTT and reinstatement of cocaine seeking are depicted in Table 1. Whereas cocaine increased both the number of premature responses in the 5-CSRTT and active responses during the reinstatement test, these effects were not correlated within individuals [Table 1; Fig. 5a; *R* = −0.046, NS]. However, the reductions in accurate choice induced by cocaine correlated with its effects on active cocaine-seeking responses in the reinstatement test [*R* = −0.46, *p* = 0.028]. Also, the increase in the number of omissions in the 5-CSRTT correlated with active cocaine-seeking responses during reinstatement [*R* = 0.62, *p* = 0.002]. Moreover, the effects of cocaine on speeding reaction times correlated with active responding in the reinstatement test [*R* = −0.48, *p* = 0.021].

**Table 1:** Correlation between cocaine- and yohimbine-induced behavioral effects compared to their respective vehicle on 5-CSRTT performance and reinstatement of cocaine seeking

	ΔCocaine active	ΔCocaine inactive	ΔYohimbine active	ΔYohimbine inactive
ΔPremature responses	−0.046	−0.13	0.058	0.22
ΔPerseverative responses	−0.22	0.38	−0.048	−0.12
ΔAccurate choice	−0.46*	−0.052	−0.070	−0.10
ΔOmissions	0.62*	0.034	0.13	0.34
ΔCorrect latency	−0.48*	0.040	−0.11	0.098
ΔFeeder latency	−0.21	0.10	0.20	0.35

Indicated are *R* values

*p < 0.05

Similar to cocaine, yohimbine-induced premature responding in the 5-CSRTT did not correlate with its effects on the reinstatement of cocaine seeking [Table 1; Fig. 5b; *R* = 0.058, NS] and neither did any of the other 5-CSRTT parameters.

## Discussion

The present data show that daily intake of cocaine transiently alters impulsive action and measures of attention in the 5-CSRTT. Furthermore, acute challenges with both cocaine and the pharmacological stressor yohimbine were found to enhance impulsive action as well as relapse to cocaine seeking, yet these effects showed no individual correlations.

During the course of the entire cocaine SA protocol, rats were concurrently trained in the 5-CSRTT, thereby allowing the assessment of shifts in baseline impulsive action and attentional functioning. Importantly, 5-CSRTT training took place in the mornings about 1 h prior to cocaine SA training and thus impulsive action was measured under drug-free conditions.

In the present study, long 5 h access to cocaine induced a transient change in impulsive action with a decrement in the number of premature responses over the weeks of cocaine self-administration. Vice versa, during extinction training, premature responding increased, yet only during the last week of extinction training. As such, the current findings extend previous observations. In this respect, Winstanley and co-workers showed that rats developed a rapid tolerance to the cocaine-induced enhancing effects on premature responding in the 5-CSRTT, whereas withdrawal from cocaine SA resulted in a rapid increase in premature responding (Winstanley et al. 2009). In contrast, no alterations of premature responding were reported earlier during periods of withdrawal in between periods of chronic cocaine self-administration (Dalley et al. 2005). An explanation for these discrepancies might lie in the amount of daily cocaine intake. In the study by Dalley and co-workers, cocaine intake was the highest (8-h sessions, 0.25 mg/infusion), compared to 2-h sessions and 0.5 mg/kg/infusion in the study of Winstanley et al. (2009). Our study, with 5-h sessions and 0.5 mg/kg/infusion would lead to a daily cocaine intake somewhere in between. This would explain the rapid (Winstanley), delayed (present study), and absence of increase in premature responding in the task (Dalley; not detectable during the 7-day withdrawal test phase).

In the current study, some measures in the 5-CSRTT, reflecting attentional performance and motivation (omissions and latencies, but not accurate choice) showed a clear deterioration over the weeks of cocaine SA which recovered to baseline performance during the extinction period. The previous observations that 2 h cocaine access only mildly affected omission rate (Winstanley et al. 2009), whereas abstinence from longer (5 and 8 h) access to cocaine more severely increased omission rates (Dalley et al. 2005), may again indicate that cocaine exposure transiently deteriorates behavioral performance depending on the intake levels of cocaine, which is restored following abstinence.

We found that within the same individuals, acute injections of both cocaine and yohimbine induced an increase in impulsive action as well as an increase in cocaine-seeking responses in the reinstatement tests. However, these drug effects on both behaviors did not correlate with each other within individuals. Thus, although cocaine and yohimbine both induced an increase in impulsive action and drug-seeking behavior, the lack of correlation suggests that the pharmacological effects of stress and a cocaine prime on relapse to cocaine seeking appear not mediated via alterations in “state” impulsive action. Although we did not study the underlying neural mechanisms of cocaine and yohimbine, the current data indicate a different mechanism of action of both cocaine and yohimbine in impulsive action and relapse to cocaine seeking. This notion is supported by previous work. For example, it has been shown that the effects of yohimbine on impulsive action in the 5-CSRTT are primarily mediated via the orbitofrontal cortex and not the medial prefrontal cortex nor the nucleus accumbens (Sun et al. 2010). In contrast, the medial prefrontal cortex and nucleus accumbens do play an important role in stress-induced reinstatement to drug-seeking behavior (for a review, see Shaham et al. 2003). In addition, both cocaine-induced increments in impulsive action as well as cocaine-primed reinstatement of drug seeking can be attenuated by a serotonin 2A antagonist suggesting a common mechanism for this receptor subtype, yet blocking serotonin 2C receptors differentially affected these behavioral processes (Fletcher et al. 2002, 2011). Together, such observations question the viability of ameliorating relapse vulnerability via a reduction of impulsive action, and these data extend our previous work on the relationship between impulsive choice, a different modality of impulsivity, and cocaine seeking (Broos et al. 2012a). In that study, both impulsive choice (delayed reward task) and relapse propensity (context-induced relapse) were manipulated with the clinically relevant drug methylphenidate and the dopamine D1 receptor antagonist SCH-23390. Similar to the current observations, we also found no correlation between the changes in impulsive choice and relapse propensity (Broos et al. 2012a). Thus, whereas acute pharmacological challenges modulate both impulsive action and impulsive choice, as well as relapse propensity, the current preclinical evidence suggest that these effects are not correlated within individuals.

These preclinical data are in line with the observed high comorbidity of ADHD and drug dependence in humans, yet the role of ADHD (and its symptomatology) in the etiology of drug dependence is unclear (Lynskey and Hall 2001). Moreover, medication improving ADHD symptoms such as, for instance, methylphenidate does not concurrently attenuate drug craving or relapse to cocaine consumption (Schubiner et al. 2002; for a review, see Pattij and De Vries 2013). This might imply that rehabilitation programs aimed at acute, short-term reduction of impulsivity will not suffice to maintain treatment retention.

In conclusion, the current observations demonstrate that a history of cocaine consumption alters impulsive action and measures of attention. Importantly, drug-induced increments in state impulsive action appear not to be associated with increments in relapse propensity within the same individuals. Collectively, this may imply that the treatment potential of promoting abstinence via reducing state impulsivity is not straightforward and warrants further investigation.
